# Risk of lung cancer and renin–angiotensin blockade: a concise review

**DOI:** 10.1007/s00432-020-03445-x

**Published:** 2020-11-24

**Authors:** Tobias Rachow, Helmut Schiffl, Susanne M. Lang

**Affiliations:** 1 Klinik Für Innere Medizin II, Sektion Pneumologische Onkologie, Universitätsklinikum Jena, Am Klinikum 1, 07747 Jena, Germany; 2 Department of Internal Medicine IV, University Hospital LMU Munich, Munich, Germany

**Keywords:** Renin–angiotensin–aldosterone system, Angiotensin enzyme inhibitors, Angiotensin receptor blockers, Non-small cell lung cancer

## Abstract

**Purpose:**

The blockade of the renin–angiotensin–aldosterone system (RAAS) by angiotensin-converting enzyme inhibitors (ACEIs) or angiotensin receptor blockers (ARBs) is one of the most common treatments for hypertension, heart failure and renal diseases. However, concerns have been raised about a possible link between RAAS-blockers and an increased risk of cancer, particularly of lung cancer. This narrative review aims to give a critical appraisal of current evidence and to help physicians understand potential links between RAAS blockade and de novo lung cancer development.

**Methods:**

Numerous pharmaco-epidemiologic studies, mostly retrospective cohort analyses, evaluated the association of RAAS blockade with lung cancer incidence and reported inconsistent findings. Meta-analyses could not further clarify a possible link between RAAS blockade and the risk of lung cancer.

**Results:**

International regulatory agencies (FDA, EMA) have concluded that the use of RAAS blockers is not associated with an increased risk of developing lung cancer. Co-administration of RAAS blockers to systemic therapy of advanced non-small cell lung cancer seems to have positive effects on the outcome.

**Conclusion:**

Until more comprehensive analyses have been completed, there is no need to change clinical practise. Additional prospective randomized trials with long-term follow-up are needed to investigate the effects of these drugs on the development and progression of lung cancer.

## Introduction

The blockade of the renin–angiotensin–aldosterone system (RAAS) has immensely improved the treatment of patients with chronic arterial hypertension, acute myocardial infarction, chronic systolic heart failure, stroke, diabetic nephropathy and other chronic kidney diseases. Angiotensin-converting enzyme inhibitors (ACEIs) and angiotensin II receptor type 1 antagonists (ARBs) represent the two most widely used RAAS blockers in clinical practice. They share similar indications and contraindications but differ in the way they affect the RAAS.

The significant benefits and risks of drugs blocking the RAAS have been documented in numerous randomized trials involving thousands of patients. The other side of the coin, however, is the concern about a possible link of the RAAS blockade and the incidence, progression and mortality of solid malignancies, particularly of lung cancer (George et al. 2010). However, well published pharmaco-epidemiological studies reported confusing results and found either an increased risk, or decreased occurrence, or no association of ACEIs or ARBs with cancer or lung cancer (Cronin-Fenton 2018).

The uncertainty regarding the safety of ACEIs or ARBs may put patients at an increased risk of cancer. A potential causative association between RAAS blockade and cancer could ban the use of these effective and well-tolerated therapeutic agents in millions of people worldwide.

The primary purpose of this narrative review is to analyse the published literature, to discuss a potential causative association of RAAS blockade and risk of lung cancer, and to determine whether this conceivable adverse effect mandates changes in current clinical practice. Second, we review the potential clinical effects of the RAAS blockade on the progression of advanced lung cancer in patients receiving systemic therapy.

## Biological links between the RAAS and lung cancer

**Fig. 1:**
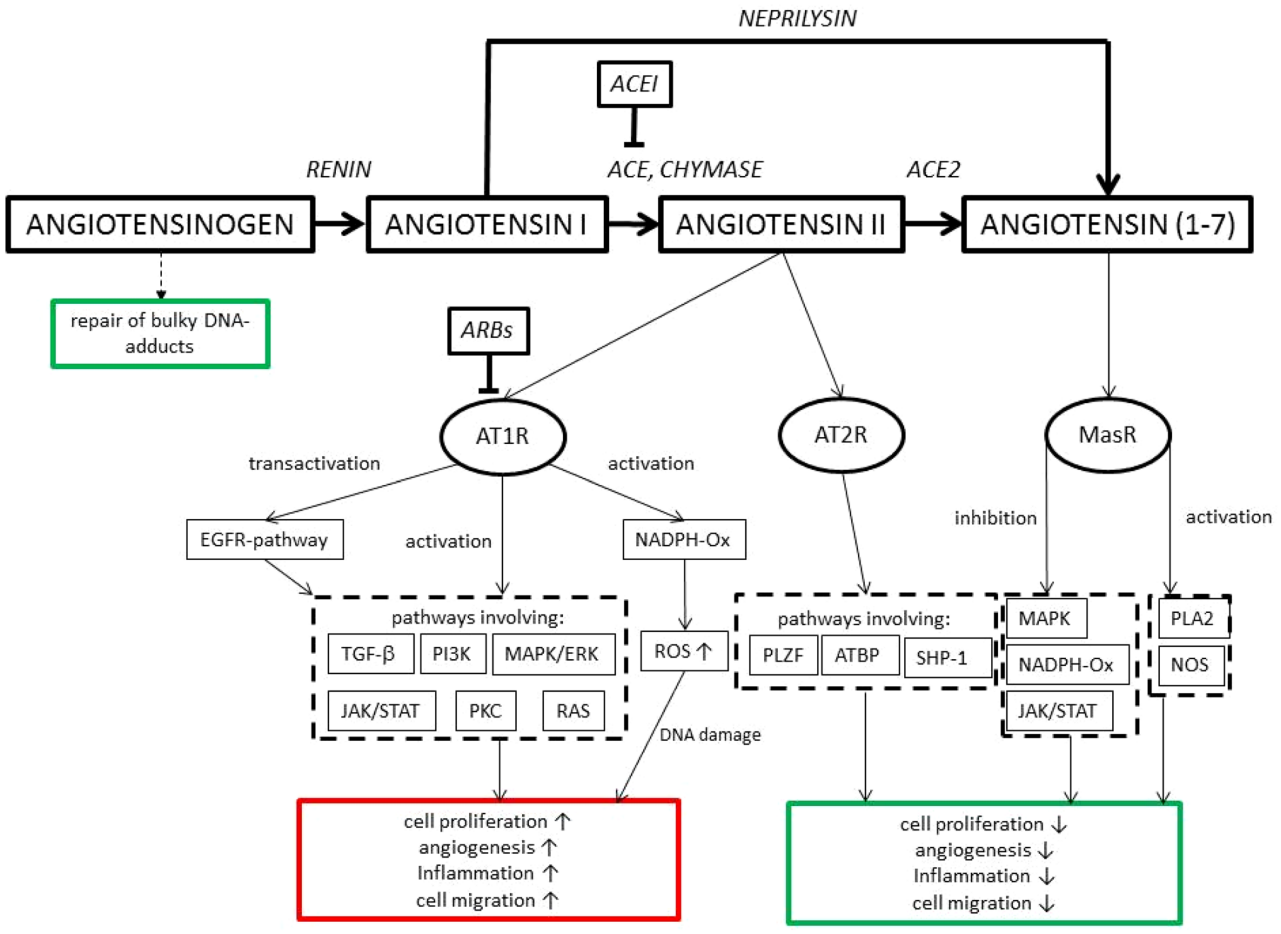
Simplified scheme of the RAAS cascade and signal transduction pathways in carcinogenesis. *ACEI* angiotensin converting enzyme inhibitor, *ACE* angiotensin converting enzyme, *ACE2* angiotensin converting enzyme-2, *ARBs* angiotensin receptor blockers, *AT1R* angiotensin-II-receptor type 1, *AT2R* angiotensin-II-receptor type 2, *MasR* Mas receptor, *EGFR* epidermal growth factor receptor, *NADPH-Ox* nicotinamide adenine dinucleotide phosphate oxidase, *ROS* reactive oxygen species, *TGF-beta* transforming growth factor beta, *PI3K* phosphoinositide 3-kinase, *MAPK/ERK* mitogen-activated protein kinase/extracellular signal-regulated kinase, *JAK* Janus kinase, *STAT* signal transducer and activator of transcription, *PKC* protein kinase C, *RAS* rat sarcoma protein, *PLZF* promyelocytic leukemia zink finger protein, *ATBP* AT2R binding protein, *SHP-1* Src homology region 2 domain-containing phosphatase-1, *PLA-2* phospholipase 2, *NOS* nitric oxide synthase

Experimental studies provide a scientific foundation for the role of the RAAS and its blockade in the regulation of cell proliferation, tissue invasion and migration, angiogenesis and tumour progression (Ishikane and Takahashi-Yanaga 2018). There is a plethora of experimental data implicating the RAAS system in signal transduction, DNA repair and angiogenesis (Fig. 1).

Angiotensinogen gene expression is upregulated in tumor samples compared to normal lung tissues. It plays a role in the repair of smoking-induced bulky DNA adducts (Goldstein et al. 2017; Sedgwick 2004). The protease renin catalyses the cleavage of angiotensinogen to angiotensin I. Its precursor protein prorenin mediates intracellular processes in tumour cells by activating the WNT/beta-catenin-pathway, which is deregulated in many solid cancers (Zhan et al. 2017). Angiotensin I is subsequently cleaved by angiotensin I converting enzyme (ACE) to angiotensin II. The exact mechanisms of angiotensin II-mediated carcinogenesis are unknown. Angiotensin II acts via two distinct transmembrane receptors: AT1R and AT2R. Particularly AT1R is implicated in lung cancer carcinogenesis. The production of endothelial adhesion molecules [E-selectin, P-selectin, intercellular adhesion molecule 1 (ICAM-1), vascular cell adhesion molecule 1 (VCAM-1)] is upregulated by activation of AT1R (Alvarez et al. 2004). Furthermore, the epidermal growth-factor receptor (EGFR) is transactivated by AT1R (Greco et al. 2003). Several molecules which are linked to inflammatory processes are induced by AT1R (Suzuki et al. 2003; Tsutamoto et al. 2000). Furthermore, angiotensin II increases the cancer stem cell-like phenotype in human non-small cell lung cancer cell lines, which has been shown to promote cancer progression, metastasis and chemotherapeutic resistance (Tawinwung et al. 2015).

Levels of vascular endothelial growth factor (VEGF) are increased by both, AT1R and AT2R. Angiogenesis is an essential process for the survival and growth of solid neoplasms and can be successfully targeted in many solid cancers. Arrieta et al. showed an association of poor prognosis and high AT1R expression in astrocytoma—a tumor type with intense vascular proliferation (Arrieta et al. 2008). AT1R overexpression and increased microvessel density were also shown in bladder cancer (Shirotake et al. 2011). In an AT1R deficient mice model tumor growth was significantly inhibited compared to mice with wildtype AT1R (Egami et al. 2003). While AT1R promotes carcinogenesis, the role of AT2R is less clear. Adenoviral mediated overexpression of AT2R on bladder cancer cells and xenograft tumor models of bladder cancer significantly reduced xenograft growth and tumor angiogenesis (Pei et al. 2017). This data indicate that the activation of both receptor types may have different effects on tumor growth.

Increased ACE concentrations are well-established biomarkers in a series of diseases affecting the lungs. In lung tumour tissues, however, ACE concentrations were found to be reduced (Danilov et al. 2019). The lowest serum ACE levels correlated with poor prognosis and higher relapse rates as well as with metastatic disease (Romer 1981). One explanation is that pulmonary vascular endothelium is the main metabolic site for angiotensin I-converting enzyme (ACE)-mediated protein degradation and the primary source for circulating ACE. The decrease in serum ACE levels might reflect an increased tumor burden as malignant cells destroy these pulmonary epithelial cells. Accordingly, plasma ACE levels may have a role as prognostic biomarkers in lung cancer (Varela and Bosco Lopez Saez 1993). ACE2 has recently gained attention in the COVID-19 pandemic (Vaduganathan et al. 2020). It has been reported that overexpression of angiotensin II-converting enzyme (ACE2) inhibits lung cancer proliferation and angiogenesis (Feng et al. 2011). It is noteworthy that polymorphisms in the ACE gene are significantly different between Asian and Caucasian populations (Li et al. 2012).

ARBs lead to the decreased activation of AT1R only, while ACEIs inhibit the formation of angiotensin II and lead to a decreased activation of both receptors. ACEIs may lead to an accumulation of bradykinin and substance P in the lung, which have been associated with tumour proliferation and angiogenesis (Hicks et al. 2018).

## RAAS blockade and risk of lung cancer

### Meta-analyses

Sipahi et al. observed a modestly increased risk of cancer associated with the use of ARBs (7.2% versus 6.0% in controls; *p* = 0.016) (Sipahi et al. 2010). The authors used aggregate data of five randomized controlled trials (RCTs). Most patients (85.7%) received telmisartan as the study drug. The analysis of solid organ cancers (breast, prostate, lung) revealed a significantly higher occurrence of new lung cancer in patients receiving ARBs (0.9% vs 0.7% in controls; RR = 1.25; *p* = 0.01), but there was no difference in death from cancer. There are limitations specific to the meta-analysis performed by Sipahi et al., which should be considered when interpreting these results. The duration of follow-up in the trials included in this meta-analysis ranged from 1.9 to 4.8 years. Because cancer is relatively rare in any time period of less than 5 years, it has been argued that the duration of these RCTs was too short to draw any meaningful conclusions about the development of new cancers. In addition, the occurrence of cancer is a relatively rare adverse event (AE), and rare AEs are often not statistically analysed in RCTs because of the small sample size. This problem persists even when data are pooled. It is also important to note that the results of this meta-analysis are based on post-hoc analyses, and the primary trials were not designed to draw conclusions about a possible class effect for all ARBs because the overwhelming majority of patients received telmisartan. As noted by the authors, publication bias was also a significant limiting factor in this meta-analysis. Specifically, many large trials did not collect or provide data on cancer incidence to the authors of this meta-analysis. Data on cancer incidence and/or cancer death were only available from 9 out of 60 trials identified as meeting the inclusion criteria for this meta-analysis.

The results of subsequent meta-analyses refuted the results of the Sipahi analysis. The meta-analysis reported by Bangalore et al. identified 70 randomized controlled trials with 324,168 participants (Bangalore et al. 2011). The authors recorded no difference in the risk of cancer with ARBs, ACEIs or other common antihypertensive drugs versus placebo. No differences were detected in cancer-related mortality for ARBs or ACEIs or other antihypertensive drugs, and the combination of ACEIs plus ARBs. In addition, results did not differ for telmisartan compared with other ARBs.

The ARB Trialists Collaboration assessed the effects of individual ARBs (candesartan, irbesartan, telmisartan, valsartan, and losartan) for incident cancers in 15 multicenter double-blind RCTs of these agents involving 138,769 participants with a high cardiovascular disease risk (Collaboration 2011). In this analysis, the RCTs included were required to have an average follow-up of at least 12 months. The authors state that there was no excess overall or site-specific cancer incidence (lung, breast, and prostate) with ARB therapy compared to controls. This analysis also examined the cancer risk of ARB/ACEI combination therapy vs ACEI, ARBs versus ACEIs, and ARBs vs placebo/controls. No increased risk of cancer was observed in any of these overall comparisons. A small Chinese meta-analysis included 8 trials and found a reduced risk for lung cancer in ARB users compared to non-ARB users (Zhang et al. 2015).

Bangalore’s and the ARB Trialists’ meta-analyses were more robust than the Sipahi meta-analysis because they included more trials and performed multiple comparison analyses. Nevertheless, information on cancer rates in individual RCTs was incomplete. Bangalore et al. acknowledged several limitations including the possibility that the survival benefit associated with antihypertensive pharmacotherapy compared with placebo may have introduced a survival bias which increased the incidence of cancer in active treatment groups. It is true for all meta-analyses that some confounding variables are nearly impossible to measure, such as exposure to radiation or carcinogens. Furthermore, none of these meta-analyses took the incidence of a specific cancer in the general population into consideration. Another limitation of meta-analyses is the selection criteria used to include trials, as the selection of trials may influence the findings (i.e. certain trials when put together could increase, decrease, or have no effect on cancer risk). As mentioned before, results are limited by the short-term nature of most trials and the short duration of exposure to the drugs in question to truly determine cancer risk.

Recently, Datzmann et al. conducted a systematic review and a meta-analysis with 12 publications with available study data on ARBs and carcinogenicity as primary outcome (Datzmann et al. 2019). The authors identified seven RCTs, four case–control studies and one cohort study focusing only on high evidence levels. Their conclusions were that there is no relationship between the use of ARBs and an increased risk of lung cancer.

In conclusion, the meta-analyses could not convincingly show or disprove an effect of the use of ARBs or ACEIs on the incidence of lung cancer.

### Population-based studies

Population-based studies have evaluated the association between drugs blocking the RAAS and cancer. The reported data show inconsistent effects (Table 1).

**Table 1:** Effects of ARB or ACEI use on lung cancer risk

References	Population	Number of patients	Time period Follow-up	Inclusion criteria	RAAS blocker	Effect
Lever et al. (1998)	Registrar General Scotland database East of Scotland Cancer Registry	5207	1980–1995 follow-up 6,6 (SD 4.5) years	Hypertension treated in the clinic	ACEIs, all cancers	Reduced risk
					ACEI, Smoking related cancers	Reduced risk
					Other antihypertensive drugs, all cancers	No effect
Friis et al. (2001)	Prescription Database of North Jutland County and the Danish Cancer Registry	17,897	1989–1995 follow-up 3.7 years (range 0–8)	Prescriptions of ACEI	ACEI, lung cancer, all cancers	No effect
					ACEI vs beta-blockers or calcium channel blockers, all cancers, lung cancer	No effect
Chang et al. (2011)	Taiwan National Health Insurance claims database	21,750	2000 median follow up 7.4 years	New diabetic patients	ARBs: all, all cancers	No effect
					ARBs: Losartan, all cancers	Reduced risk
					ARBs: Candesartan and telmisartan, all cancers	Increased risk
Huang et al. (2011)	Taiwan National Health Insurance Research Database	109,002 40,124 ARB users 68,878 controls	1998–2006 Mean follow-up 5.7 (SD 2.6) years	New hypertensive patients	ARB vs controls, all cancers	Reduced risk
Pasternak et al. (2011)	Danish registries on filled drug prescription	107,466 ARB 209,692 ACEI	1998–2006 Follow-up ARB users 140,562 person-years ACEI users 163,617 person-years	New ARB/ACEI users	ARB/ACEI combined, all cancers, lung cancer	No effect
Azoulay et al. (2012)	UK General Practice Research Database	1,165,781	1995–2010 Mean follow-up 6.4 (SD 3.9) years	New diagnosis of lung, colorectal, breast and prostate cancer	ARBs vs diuretics/ß-blockers, all cancers, lung cancer	No effect
					ACEIs vs diuretics/ß-blockers, lung cancer	Increased risk
Bhaskaran et al. (2012)	UK General Practice Research Database	377,649 340,589 ACEI 37,060 ARB	1995–2010 median follow-up 4.6 years	New ACEI or ARB users with at least one year of initial treatment	Combined ACEI/ARB, lung cancer	Reduced risk
					Combined ACEI/ARB, all cancers	No effect
Rao et al. (2013)	Department of Veterans Affairs electronic medical record system and registries	1,229,902	1999–2010 mean follow-up 4.5 (SD 2.1) years	New ARB dispensation Controls = randomly identified patients in 1:15 ratio	ARB vs controls, lung cancer	Small risk reduction
Chiang et al. (2014)	Taiwan National Health Insurance Research Database	6,960 ARB 4,988 ACEI 143,887 non ACEI/ARB	2000–2008 Mean follow-up ACEI 2.36 (SD1.81) ARB 2.38 (SD1.77) years	Essential hypertension no COPD no cancer	ARB vs control, all cancers	Reduced risk
					ACEI vs control, all cancers	Reduced risk
Gokhale et al. (2016)	20% sample of the Medicare claims	108,116 ARB 342,611 ACEI	2007–2012 median follow-up 0.7 years	New ARB/ACEI users	ARBs, lung cancer	No effect
					ACEIs, lung cancer	No effect
Tascilar et al. (2016)	UK Clinical Practice Research Datalink	60,109	2000–2008 mean follow-up 3.9 (SD 2.2) years	New ARB users	Telmisartan compared to other ARBs, lung cancer, all cancers	No effect
Hicks et al. (2018)	United Kingdom Clinical Practice Research Datalink	992,061 335,135 ACEI 29,008 ARB 101,637 both ACEIs and ARBs	1995–2015 mean follow-up 6.4 (SD 4.7) years	New antihypertensive drug users	ACEI vs ARB	Increased risk
					Cumulative duration of ACEI use	Increasing risk with longer duration (≤ 5; 5–10; > 10 Years)
Lin et al. (2020)	National Health Insurance Research Database (NHIRD) and Taiwan Air Quality Monitoring Database (TAQMD)	22,384 ACEI 22,384 ARB	2000–2012 mean follow-up: 6.33 ± 3.52 years ARB 6.12 ± 3.47 years ACEI	New ARB/ACEI users No known history of cancer	ACEIs vs ARBs, lung cancer	Increased risk

*ACEI* ACE inhibitors, *ARB* angiotensin receptor blockers, *SD* standard deviation

After the introduction of ACE inhibitors in Scotland in 1980 a first cohort study was reported by Lever et al. indicating a risk reduction of the incident (RR 0.72) and fatal (RR 0.65) cancer among 1559 patients receiving ACEIs (Lever et al. 1998).

In subsequent studies, there was a reduced lung cancer risk of ARBs and/or ACEIs in some cohorts(Bhaskaran et al. 2012; Chiang et al. 2014; Huang et al. 2011; Rao et al. 2013) and no effect of ARBs and/or ACEIs in others, (Friis et al. 2001; Gokhale et al. 2016; Pasternak et al. 2011; Tascilar et al. 2016). Azoulay et al. observed an increased risk for ACEIs but not for ARBs (Azoulay et al. 2012) and Chiang noted mixed results for individual ARBs (Chang et al. 2011).

The two most recently published studies showed an increased risk of lung cancer. In Caucasian patients from a large UK based cohort there was an association between ACEI use and lung cancer with an overall 14% increased risk of lung cancer, evident after 5 years of use and increasing with longer duration of at least 10 years (Hicks et al. 2018).

The most recent study from Taiwan combined data from the National Health Insurance Research Database (NHIRD) and the Taiwan Air Quality Monitoring Database (TAQMD) to determine lung cancer risk in Asian ACEI users in comparison to a propensity score-matched group of ARB users. They found a significantly higher risk of lung cancer in the ACEI cohort than in the ARB cohort (HR 1.36; 95% CI 1.11–1.67) and also a dose–response relationship with a higher lung cancer rate in patients with more intensive treatment as compared to those with little or no exposure to ACEIs.

## Critical appraisal

The pharmaco-epidemiological cohort studies examining the risk of cancer, particularly of lung cancer associated with drugs blocking the RAAS have produced a data jungle. This may be attributable to the fact that even the most well designed and carefully conducted observational studies have limitations and are prone to residual confounding and bias. Observational studies can only show associations, but do not prove an actual cause and effect relationship and caution should be used when data from non-randomized cohort studies are evaluated.

Another scientific approach is the analysis of big data from a real-world setting rather than a study setting. Big health care databases can be used for nationwide studies including large numbers of subjects. However, big data have their own drawbacks such as unreliable and incomplete data sets. A common technique used to analyse big data is to link one variable (ARB use or ACEI use) to another variable (cancer). However, these associations may not always stand for substantial or meaningful findings. Lung cancer is a multifactorial disorder. Its development may be favoured by numerous factors. Major causes are primarily smoking and exposure to air pollution, occupational carcinogens and genetic disposition. Obesity, alcohol consumption, age and gender may also increase cancer risk. Amazingly, all cohort studies included smokers and ex-smokers, but information on the onset of smoking, pack years or quitting smoking is dubious or lacking. Only the most recent study reported environmental exposure but none of the analyses included exposure to radiation, chemicals or other carcinogens (Lin et al. 2020).

A further weakness is a fact that most studies were designed to assess the overall risk of cancer and not of lung cancer specifically, resulting in small cohorts. Several studies had methodological shortcomings, including short duration of follow up, failure to account for clinically inapparent cancer, and the inclusion of prevalent users of drugs affecting the RAAS. Another factor that confounds the interpretation of the presented data is the lack of standardized screening for lung cancer. A surveillance bias may be found in patients receiving ACEIs compared to ARBs or other antihypertensive drugs. ACEI users are more likely to develop cough and to have more opportunities for a diagnostic work-up by their physician. In the discussion of the Asian data the frequency of cough of > 50% in Chinese patients taking ACE inhibitors, leading to a higher rate of tumor detection, should be kept in mind (Huang et al. 2011).

Even the study by Hicks et al., using rigorous analytical approaches had serious limitations. There were more obese patients (32.3 vs 19.9%), more people with alcohol problems (8.7% vs 6.8%) and less never-smokers (47.9% vs 49%) in the patient group receiving ACEIs than in the control group. In addition, the ACEI group was older (57.8 years vs 54.9 years) and more participants were male (57.8 vs 54.9 years). Moreover, socioeconomic differences (not analysed) might have influenced prescribing patterns and lung cancer risk over a long time period in the UK national health care setting or other health care systems. Other limitations of this investigation or other studies are the lack of information of the drug dosage or non-adherence to the prescribed drugs, both might bias the risk of lung cancer.

Current evidence is inadequate to establish a causal relationship between RAAS blocker use and development of cancer, particularly of lung cancer. The number, size and quality of randomized trials are too low to provide conclusive evidence. The situation has not been helped by the seemingly contradictory findings of the studies analysed in this review. Overestimation of quantitatively small effects observed by low-quality pharmacoepidemiologic studies led to much of the unnecessary controversy. The association of RAAS blocker use and increased lung cancer risk is unlikely monocausal.

### Statement of regulatory agencies

The surveillance of drug safety is of paramount importance and was closely monitored in all drug trials. The data from clinical studies regarding efficacy and safety are carefully reviewed by international regulatory agencies. The rigorous analysis performed by the Food and Drug Administration and the European Medicines Agency has indicated no signal of harm associated with ARB or ACEI therapy in patients with incident cancers.

## RAAS blockade and clinical outcome of lung cancer patients

Numerous clinical studies have revealed that RAAS blockers may have beneficial effects on a broad range of malignancies. The gain in overall survival varies with tumour type and stage, but the response to RAAS blockade may also depend on certain tumour characteristics, cancer treatment, the class of RAAS blocker used and its dosage. Non-small cell lung cancer (NSCLC) seems to belong to the responsive tumor type (Pinter and Jain 2017).

In patients with advanced NSCLC treated with chemotherapy, tyrosine kinase inhibitors or immunotherapy, there was no observation in any of the retrospective studies that RAAS-blockers may have a detrimental effect (Table 2) (Aydiner et al. 2015; Menter et al. 2017; Miao et al. 2016; Wang et al. 2017; Wei et al. 2019; Wilop et al. 2009). On the contrary, clinical evidence from these retrospective trials indicates that RAAS blockers may have beneficial effects such as improved survival, particularly in late stages of lung cancer.

**Table 2:** Effects of ARB or ACEI use on lung cancer treatment outcome

References	Design	Number of patients	Time period	Inclusion criteria	Results	
Wilop et al. (2009)	Retrospective analysis	287	1996–2007	Long-term medication with ACEI or ARB advanced NSCLC (Stage IIb or IV) undergoing first-line platinum-based chemotherapy	52 ACEI/ARB (43/9) vs 235 non ACEI/ARB	OS 11.7 vs. 8.6 months, HR 0.56, * p* = 0.03
Aydiner et al. (2015)	Retrospective analysis	37 ACEI/ARB 80 non ACEI/ARB Matched 1:2	2003–2011	NSCLC stage IV Chemotherapy or Erlotinib	ACEI/ARB overall	HR 0.99 (95%CI 0.49–2.00) * p* = 0.98
					ACEI/ARB + Erlotinib	HR 0.37 (95%CI 0.17–0,76) * p* = 0.008
Miao et al. (2016)	Retrospective analysis	228 advanced NCCLC 73 early-stage disease	2000–2014	Histologically confirmed advanced NCCLC (stage IIIb or IV) or early-stage disease (stage I-IIIa) and platinum-based chemotherapy	112 TKI ± Chemotherapy 18 ACEI/ARB vs 94 non-ACEI/ARB	PFS 11.2 vs 8.0 months (*p* = 0.044) OS not significant
					228 advanced NSCLC 38 ACEI/ARB vs 190 non-ACEI/ARB	PFS 7.3 vs 5.2 months (*p* = 0.036) OS not significant
					73 early stage /surgery 14 ACEI/ARB vs 59 non-ACEI/ARB	PFS not significant OS not significant
Menter et al. (2017)	Retrospective analysis	1813 patients 273 of 1,465 CP had ACEI/ARB 78 of 348 had CBP + ACEI/ARB	2005–2011	patients with advanced NSCLC concomitant RAAS blocker treatment during 1L CP without or with bevacizumab (CPB)	CP with vs. without concomitant ACEI/ARB	Median OS 12.9 vs 8.4 months (crude HR 0.72, 95% CI 0.63–0.84) statistically significant
					CPB with vs. without concomitant ACEI/ARB	Median OS was 14.9 vs 11.9 months (crude HR 0.77, 95% CI 0.57–1.02) not statistically significant
Wang et al. (2017)	meta-analysis	9 studies 29,156 patients	All studies until 02/2017	Lung cancer ARBs/ACEIs usage	Lung cancer patients	OS: pooled HR 0.86 (95% CI: 0.76–0.98, * p* = 0.022) PFS: no demonstrable impact
					NSCLC vs all types of lung cancer	HR 0.78 (95% CI, 0.65–0.93) vs HR 0.96 (95% CI, 0.81–1.13) * p* = 0.005
					Advanced clinical stage IIIb to IV	HR 0.77 (95% CI, 0.64–0.92)
					ACEIs usage group vs ARBs usage group	OS: pooled HR 0.83 (95% CI, 0.53–1.30) and 0.95 (95% CI, 0.74–1.22)
Wei et al. (2019)	Retrospective cohort	678 Chinese patients ACEI * n* = 97, ARB * n* = 117 non-ACEI/ARB * n* = 461	2016–2018	Patients using anti-hypertensive drugs at least six months before the first diagnosis of lung cancer	Chemotherapy (*n* = 117) 38 ACEI/ARB (32.5%) vs 79 non-ACEI/ARB (67.5%)	PFS 10.7 (ACEI/ARB) vs. 6.7 months (non-ACEI/ARB), * p* = 0.040 OS no significant difference
					ACEI (*n* = 20) vs non-ACEI/ARB (*n* = 76)	PFS (12.9 vs 6.4, * p* = 0.014)
					ARB (*n* = 18) vs non-ACEI/ARB (*n* = 76)	PFS (6.7 vs 6.4, * p* = 0.581)

*ACEI* ACE inhibitors, *ARB* angiotensin receptor blockers, *CP* carboplatin and paclitaxel, *CPB* carboplatin and paclitaxel and bevacizumab, *TKI* tyrosine kinase inhibitor, *OS* overall survival, *PFS* progression free survival, *HR* hazard ratio, *CI* confidence interval

## Critical appraisal

Currently, there is no large randomized controlled trial addressing potential effects of ACEIs or ARBs on the survival of lung cancer patients undergoing systemic treatment. There are few retrospective studies with a small number of subjects included and a large variation in treatments received demonstrating mostly a positive effect of RAAS blockade. None showed a negative effect (Table 2). This is in congruence with data from experimental studies suggesting an influence of the RAAS blockade on cellular signalling, angiogenesis and tumour growth (Pinter and Jain 2017). However, the generalizability of the data presented is dubious. There are pharmacogenomic and tumour genetic differences in Chinese and Caucasian patients which need to be considered when evaluating response to anticancer agents.

Whether or not a patient with lung cancer died from lung cancer or from cardiovascular events remains uncertain. The effects observed may be due in part to the prevention of cardiovascular events by ACEIs and ARBs. At present, with the limited data available, these findings should be considered as hypothesis-generating.

## Conclusions

There is a vast number of analyses addressing the question whether the use of ARBs or ACEIs is associated with a higher incidence of lung cancer. The plethora of data provided no clear signal. Until more comprehensive analyses have been completed, available data do not warrant any change in current clinical recommendations of practice regarding the use of RAAS blockers.

It is interesting to note that patients with lung cancer undergoing treatment may benefit from ARBs or ACEIs. This is in accordance with basic science, demonstrating a role of the RAAS in tumour growth.

Further prospective studies (RCTs) with long-term follow up and a defined screening strategy for lung cancer are needed in patients with low risk of lung cancer to enhance the scientific evidence of the long-term safety of these drugs. RCTs should try to elucidate a possible role of RAAS blockade during systemic cancer treatment.

## References

[CR1] Alvarez A et al (2004) Direct evidence of leukocyte adhesion in arterioles by angiotensin II. Blood 104:402–408. 10.1182/blood-2003-08-2974 15044253 10.1182/blood-2003-08-2974

[CR2] Arrieta O et al (2008) Expression of AT1 and AT2 angiotensin receptors in astrocytomas is associated with poor prognosis. Br J Cancer 99:160–166. 10.1038/sj.bjc.6604431 18594540 10.1038/sj.bjc.6604431 PMC2453037

[CR3] Aydiner A, Ciftci R, Sen F (2015) Renin-Angiotensin system blockers may prolong survival of metastatic non-small cell lung cancer patients receiving erlotinib. Medicine (Baltimore) 94:e887. 10.1097/MD.0000000000000887 26039117 10.1097/MD.0000000000000887 PMC4616356

[CR4] Azoulay L, Assimes TL, Yin H, Bartels DB, Schiffrin EL, Suissa S (2012) Long-term use of angiotensin receptor blockers and the risk of cancer. PLoS ONE 7:e50893. 10.1371/journal.pone.0050893 23251399 10.1371/journal.pone.0050893 PMC3521027

[CR5] Bangalore S et al (2011) Antihypertensive drugs and risk of cancer: network meta-analyses and trial sequential analyses of 324,168 participants from randomised trials. Lancet Oncol 12:65–82. 10.1016/s1470-2045(10)70260-6 21123111 10.1016/S1470-2045(10)70260-6

[CR6] Bhaskaran K, Douglas I, Evans S, van Staa T, Smeeth L (2012) Angiotensin receptor blockers and risk of cancer: cohort study among people receiving antihypertensive drugs in UK General Practice Research Database. BMJ 344:e2697. 10.1136/bmj.e2697 22531797 10.1136/bmj.e2697 PMC3339864

[CR7] Chang CH, Lin JW, Wu LC, Lai MS (2011) Angiotensin receptor blockade and risk of cancer in type 2 diabetes mellitus: a nationwide case-control study. J Clin Oncol 29:3001–3007. 10.1200/JCO.2011.35.1908 21690476 10.1200/JCO.2011.35.1908

[CR8] Chiang YY, Chen KB, Tsai TH, Tsai WC (2014) Lowered cancer risk with ACE inhibitors/ARBs: a population-based cohort study. J Clin Hypertens (Greenwich) 16:27–33. 10.1111/jch.12228 24304931 10.1111/jch.12228 PMC8031532

[CR9] Collaboration ARBT (2011) Effects of telmisartan, irbesartan, valsartan, candesartan, and losartan on cancers in 15 trials enrolling 138,769 individuals. J Hypertens 29:623–635. 10.1097/HJH.0b013e328344a7de 21358417 10.1097/HJH.0b013e328344a7de

[CR10] Cronin-Fenton D (2018) Angiotensin converting enzyme inhibitors and lung cancer. BMJ 363:k4337. 10.1136/bmj.k4337 30355573 10.1136/bmj.k4337

[CR11] Danilov SM, Metzger R, Klieser E, Sotlar K, Trakht IN, Garcia JGN (2019) Tissue ACE phenotyping in lung cancer. PLoS ONE 14:e0226553. 10.1371/journal.pone.0226553 31877149 10.1371/journal.pone.0226553 PMC6932779

[CR12] Datzmann T, Fuchs S, Andree D, Hohenstein B, Schmitt J, Schindler C (2019) Systematic review and meta-analysis of randomised controlled clinical trial evidence refutes relationship between pharmacotherapy with angiotensin-receptor blockers and an increased risk of cancer. Eur J Intern Med 64:1–9. 10.1016/j.ejim.2019.04.019 31060961 10.1016/j.ejim.2019.04.019

[CR13] Egami K et al (2003) Role of host angiotensin II type 1 receptor in tumor angiogenesis and growth. J Clin Invest 112:67–75. 10.1172/jci16645 12840060 10.1172/JCI16645 PMC162282

[CR14] Feng Y et al (2011) Overexpression of ACE2 produces antitumor effects via inhibition of angiogenesis and tumor cell invasion in vivo and in vitro. Oncol Rep 26:1157–1164. 10.3892/or.2011.1394 21769437 10.3892/or.2011.1394

[CR15] Friis S, Sorensen HT, Mellemkjaer L, McLaughlin JK, Nielsen GL, Blot WJ, Olsen JH (2001) Angiotensin-converting enzyme inhibitors and the risk of cancer: a population-based cohort study in Denmark. Cancer 92:2462–2470. 10.1002/1097-0142(20011101)92:9%3c2462::aid-cncr1596%3e3.0.co;2-l 11745304 10.1002/1097-0142(20011101)92:9<2462::aid-cncr1596>3.0.co;2-l

[CR16] George AJ, Thomas WG, Hannan RD (2010) The renin-angiotensin system and cancer: old dog, new tricks. Nat Rev Cancer 10:745–759. 10.1038/nrc2945 20966920 10.1038/nrc2945

[CR17] Gokhale M, Girman C, Chen Y, Pate V, Funk MJ, Sturmer T (2016) Comparison of diagnostic evaluations for cough among initiators of angiotensin converting enzyme inhibitors and angiotensin receptor blockers. Pharmacoepidemiol Drug Saf 25:512–520. 10.1002/pds.3977 26860956 10.1002/pds.3977 PMC4933308

[CR18] Goldstein B, Trivedi M, Speth RC (2017) Alterations in gene expression of components of the renin-angiotensin system and its related enzymes in lung cancer. Lung Cancer Int 2017:6914976. 10.1155/2017/6914976 28791183 10.1155/2017/6914976 PMC5534309

[CR19] Greco S et al (2003) Angiotensin II activates extracellular signal regulated kinases via protein kinase C and epidermal growth factor receptor in breast cancer cells. J Cell Physiol 196:370–377. 10.1002/jcp.10313 12811831 10.1002/jcp.10313

[CR20] Hicks BM, Filion KB, Yin H, Sakr L, Udell JA, Azoulay L (2018) Angiotensin converting enzyme inhibitors and risk of lung cancer: population based cohort study. BMJ 363:k4209. 10.1136/bmj.k4209 30355745 10.1136/bmj.k4209 PMC6199558

[CR21] Huang CC, Chan WL, Chen YC, Chen TJ, Lin SJ, Chen JW, Leu HB (2011) Angiotensin II receptor blockers and risk of cancer in patients with systemic hypertension. Am J Cardiol 107:1028–1033. 10.1016/j.amjcard.2010.11.026 21256465 10.1016/j.amjcard.2010.11.026

[CR22] Ishikane S, Takahashi-Yanaga F (2018) The role of angiotensin II in cancer metastasis: potential of renin-angiotensin system blockade as a treatment for cancer metastasis. Biochem Pharmacol 151:96–103. 10.1016/j.bcp.2018.03.008 29534876 10.1016/j.bcp.2018.03.008

[CR23] Lever AF et al (1998) Do inhibitors of angiotensin-I-converting enzyme protect against risk of cancer? Lancet 352:179–184. 10.1016/s0140-6736(98)03228-0 9683206 10.1016/S0140-6736(98)03228-0

[CR24] Li YF et al (2012) Angiotensin-converting enzyme (ACE) gene insertion/deletion polymorphism and ACE inhibitor-related cough: a meta-analysis. PLoS ONE 7:e37396. 10.1371/journal.pone.0037396 22723835 10.1371/journal.pone.0037396 PMC3378563

[CR25] Lin SY et al (2020) Association between angiotensin-converting enzyme inhibitors and lung cancer-a nationwide, population-based, propensity score-matched cohort study. Cancers (Basel). 10.3390/cancers12030747 32245239 10.3390/cancers12030747 PMC7140054

[CR26] Menter AR et al (2017) Effect of angiotensin system inhibitors on survival in patients receiving chemotherapy for advanced non-small-cell lung cancer. Clin Lung Cancer 18(189–197):e183. 10.1016/j.cllc.2016.07.008 10.1016/j.cllc.2016.07.008 PMC5424707 27637408

[CR27] Miao L, Chen W, Zhou L, Wan H, Gao B, Feng Y (2016) Impact of angiotensin I-converting enzyme inhibitors and angiotensin II type-1 receptor blockers on survival of patients with NSCLC. Sci Rep 6:21359. 10.1038/srep21359 26883083 10.1038/srep21359 PMC4756359

[CR28] Pasternak B, Svanstrom H, Callreus T, Melbye M, Hviid A (2011) Use of angiotensin receptor blockers and the risk of cancer. Circulation 123:1729–1736. 10.1161/circulationaha.110.007336 21482967 10.1161/CIRCULATIONAHA.110.007336

[CR29] Pei N et al (2017) Angiotensin II type 2 receptor promotes apoptosis and inhibits angiogenesis in bladder cancer. J Exp Clin Cancer Res 36:77. 10.1186/s13046-017-0542-0 28599664 10.1186/s13046-017-0542-0 PMC5466725

[CR30] Pinter M, Jain RK (2017) Targeting the renin-angiotensin system to improve cancer treatment: implications for immunotherapy. Sci Transl Med. 10.1126/scitranslmed.aan5616 28978752 10.1126/scitranslmed.aan5616 PMC5928511

[CR31] Rao GA et al (2013) Angiotensin receptor blockers: are they related to lung cancer? J Hypertens 31:1669–1675. 10.1097/HJH.0b013e3283621ea3 23822929 10.1097/HJH.0b013e3283621ea3 PMC3879726

[CR32] Romer FK (1981) Angiotensin-converting enzyme and its association with outcome in lung cancer. Br J Cancer 43:135–142. 10.1038/bjc.1981.21 6258623 10.1038/bjc.1981.21 PMC2010507

[CR33] Sedgwick B (2004) Repairing DNA-methylation damage. Nat Rev Mol Cell Biol 5:148–157. 10.1038/nrm1312 15040447 10.1038/nrm1312

[CR34] Shirotake S, Miyajima A, Kosaka T, Tanaka N, Maeda T, Kikuchi E, Oya M (2011) Angiotensin II type 1 receptor expression and microvessel density in human bladder cancer. Urology 77:1009.e1019-1025. 10.1016/j.urology.2010.11.002 10.1016/j.urology.2010.11.002 21296393

[CR35] Sipahi I, Debanne SM, Rowland DY, Simon DI, Fang JC (2010) Angiotensin-receptor blockade and risk of cancer: meta-analysis of randomised controlled trials. Lancet Oncol 11:627–636. 10.1016/s1470-2045(10)70106-6 20542468 10.1016/S1470-2045(10)70106-6 PMC4070221

[CR36] Suzuki Y, Ruiz-Ortega M, Lorenzo O, Ruperez M, Esteban V, Egido J (2003) Inflammation and angiotensin II. Int J Biochem Cell Biol 35:881–900. 10.1016/s1357-2725(02)00271-6 12676174 10.1016/s1357-2725(02)00271-6

[CR37] Tascilar K, Azoulay L, Dell’Aniello S, Bartels DB, Suissa S (2016) The use of telmisartan and the incidence of cancer. Am J Hypertens 29:1358–1365. 10.1093/ajh/hpw095 27557862 10.1093/ajh/hpw095 PMC5863774

[CR38] Tawinwung S, Ninsontia C, Chanvorachote P (2015) Angiotensin II increases cancer stem cell-like phenotype in lung cancer cells. Anticancer Res 35:4789–4797 26254369

[CR39] Tsutamoto T et al (2000) Angiotensin II type 1 receptor antagonist decreases plasma levels of tumor necrosis factor alpha, interleukin-6 and soluble adhesion molecules in patients with chronic heart failure. J Am Coll Cardiol 35:714–721. 10.1016/s0735-1097(99)00594-x 10716475 10.1016/s0735-1097(99)00594-x

[CR40] Vaduganathan M, Vardeny O, Michel T, McMurray JJV, Pfeffer MA, Solomon SD (2020) Renin-angiotensin-aldosterone system inhibitors in patients with Covid-19. N Engl J Med 382:1653–1659. 10.1056/NEJMsr2005760 32227760 10.1056/NEJMsr2005760 PMC7121452

[CR41] Varela AS, Bosco Lopez Saez JJ (1993) Utility of serum activity of angiotensin-converting enzyme as a tumor marker. Oncology 50:430–435. 10.1159/000227224 8233282 10.1159/000227224

[CR42] Wang N, Liu J, Wang W, Qin J, Lin D (2017) The impact of Renin-angiotensin system blockers on lung cancers prognosis: a prisma-compliant systematic review and meta-analysis. Allied J Med Res 1:28–34

[CR43] Wei J et al (2019) Retrospective clinical study of renin-angiotensin system blockers in lung cancer patients with hypertension. PeerJ 7:e8188. 10.7717/peerj.8188 31844581 10.7717/peerj.8188 PMC6910116

[CR44] Wilop S, von Hobe S, Crysandt M, Esser A, Osieka R, Jost E (2009) Impact of angiotensin I converting enzyme inhibitors and angiotensin II type 1 receptor blockers on survival in patients with advanced non-small-cell lung cancer undergoing first-line platinum-based chemotherapy. J Cancer Res Clin Oncol 135:1429–1435. 10.1007/s00432-009-0587-3 19399518 10.1007/s00432-009-0587-3 PMC12160257

[CR45] Zhan T, Rindtorff N, Boutros M (2017) Wnt signaling in cancer. Oncogene 36:1461–1473. 10.1038/onc.2016.304 27617575 10.1038/onc.2016.304 PMC5357762

[CR46] Zhang J et al (2015) Angiotensin receptor blockers (ARBs) reduce the risk of lung cancer: a systematic review and meta-analysis. Int J Clin Exp Med 8:12656–12660 26550179 PMC4612864

